# Cbl-b Deficiency Mediates Resistance to Programmed Death-Ligand 1/Programmed Death-1 Regulation

**DOI:** 10.3389/fimmu.2017.00042

**Published:** 2017-01-26

**Authors:** Mai Fujiwara, Emily J. Anstadt, Robert B. Clark

**Affiliations:** ^1^Department of Immunology, University of Connecticut Health Center, Farmington, CT, USA; ^2^Department of Medicine, University of Connecticut Health Center, Farmington, CT, USA

**Keywords:** T cell regulation, tumor immunity, immune checkpoint, Cbl-b, PD-L1/PD-1, NK cells

## Abstract

Casitas B-lineage lymphoma-b (Cbl-b) is an E3 ubiquitin ligase that negatively regulates T cell activation. Cbl-b^−/−^ T cells are hyper-reactive and co-stimulation independent, and Cbl-b^−/−^ mice demonstrate robust T cell and NK cell-mediated antitumor immunity. As a result of these murine studies, Cbl-b is considered a potential target for therapeutic manipulation in human cancer immunotherapy. The PD-L1/PD-1 pathway of immune regulation is presently an important therapeutic focus in tumor immunotherapy, and although Cbl-b^−/−^ mice have been shown to be resistant to several immuno-regulatory mechanisms, the sensitivity of Cbl-b^−/−^ mice to PD-L1-mediated suppression has not been reported. We now document that Cbl-b^−/−^ T cells and NK cells are resistant to PD-L1/PD-1-mediated suppression. Using a PD-L1 fusion protein (PD-L1 Ig), this resistance is shown for both *in vitro* proliferative responses and IFN-γ production and is not associated with decreased PD-1 expression on Cbl-b^−/−^ cells. In coculture studies, Cbl-b^−/−^ CD8^+^, but not CD4^+^ T cells, diminish the PD-L1 Ig-mediated suppression of bystander naïve WT CD8^+^ T cells. Using an *in vivo* model of B16 melanoma in which numerous liver metastases develop in WT mice in a PD-1 dependent manner, Cbl-b^−/−^ mice develop significantly fewer liver metastases without the administration of anti-PD-1 antibody. Overall, our findings identify a new mode of immuno-regulatory resistance associated with Cbl-b deficiency and suggest that resistance to PD-L1/PD-1-mediated suppression is a novel mechanism by which Cbl-b deficiency leads to enhanced antitumor immunity. Our results suggest that targeting Cbl-b in cancer immunotherapy offers the opportunity to simultaneously override numerous relevant “checkpoints,” including sensitivity to regulatory T cells, suppression by TGF-β, and immune regulation by both CTLA-4 and, as we now report, by the PD-L1/PD-1 pathway.

## Introduction

Casitas B-lineage lymphoma-b (Cbl-b) is an E3 ubiquitin ligase that regulates many aspects of cell activation. Cbl-b functions in T cells to regulate the activation of several proximal T cell receptor signaling molecules, including PI3K (1–4). Cbl-b deficiency in mice (Cbl-b^−/−^ mice) leads to spontaneous autoimmunity characterized by multi-organ cellular infiltration (1) as well as increased susceptibility to induced models of autoimmunity such as experimental autoimmune encephalomyelitis (2, 5, 6). T cells from Cbl-b^−/−^ mice demonstrate hyper-reactivity (1) and co-stimulation independence (2). Moreover, we and others have shown that Cbl-b^−/−^ T cells are resistant to suppression by Tregs (7–9) and TGF-β (7, 10, 11). The potential relevance of alterations in Cbl-b in autoimmunity is also supported by the finding that single nucleotide polymorphisms in the *CBLB* gene are associated with human autoimmune diseases such as systemic lupus erythematosus (12) and multiple sclerosis (13). More recently, Cbl-b^−/−^ mice have also become a focus for the study of T cell-mediated antitumor immunity, and our laboratory and others have reported that Cbl-b^−/−^ mice are resistant to the outgrowth of spontaneous and transplantable tumors (9–11). In addition to T cell-mediated effects, it has recently been reported that Cbl-b^−/−^ mice have enhanced NK cell-mediated tumor immunity (14). As a result of these studies, Cbl-b is considered a target for therapeutic manipulation in cancer immunotherapy.

The PD-L1/PD-1 pathway is recognized as an important mechanism of immune regulation in mice and humans (15, 16). Moreover, targeting this pathway for inhibition has generated much interest as a novel therapeutic approach for enhancing tumor immunity in certain human malignancies (17–19). A number of mechanisms have been proposed for the normal PD-L1/PD-1-mediated regulation of T cells (20–22), and this includes the upregulation of Cbl-b in T cells in response to PD-L1/PD-1 signaling (23). This upregulation of Cbl-b is postulated to be required for TCR down-modulation and subsequent inhibition of T cell activation by PD-L1/PD-1 signaling (23). While these studies suggest the potential involvement of Cbl-b in the normal PD-L1/PD-1 inhibition of T cell responses, this has not been directly examined in the context of Cbl-b deficiency.

In the present study, we analyzed PD-L1/PD-1-mediated immune regulation utilizing Cbl-b^−/−^ mice. We document for the first time that Cbl-b deficiency in mice results in functional resistance of T cells and NK cells to PD-L1/PD-1-mediated regulation. Our results thus add to Cbl-b’s role in immune regulation and identify a new mechanism by which Cbl-b deficiency can lead to enhanced antitumor immunity.

## Materials and Methods

### Mice

Female C57BL/6 (WT) mice were purchased from the Jackson Laboratory (Bar Harbor, ME, USA). Cbl-b^−/−^ mice on a C57BL/6 background were a gift from Dr. H. Gu (Columbia University, New York, NY, USA). Female C57BL/6 congenic mice (CD45.1^+^) were also purchased from the Jackson Laboratory. All mice were maintained and bred under specific pathogen-free conditions in accordance with the guidelines of the UConn Health Institutional Animal Care and Use Committee (IACUC) and the Center for Comparative Medicine at UConn Health. The UConn Health IACUC has approved the protocol (protocol 101448-0919) used in these studies.

### *In Vitro* Suppression of T Cell Proliferation with the Recombinant PD-L1 Fusion Protein (PD-L1 Ig)

Splenic naïve CD8^+^ CD44^low^ cells isolated *via* positive selection by magnetic bead purification (Miltenyi Biotec, Auburn, CA, USA) from WT and Cbl-b^−/−^ mice were labeled with 2.5 µM CFSE (Molecular Probe, Eugene, OR, USA) and stimulated with 2 µg/ml of plate-bound anti-CD3 ab and 0.4 µg/ml of soluble anti-CD28 ab in the presence of 9–10 µg/ml of plate-bound control Ig or PD-L1 Ig for 72 h in 10% FCS complete RPMI 1640 in round-bottom 96-wells at 5 × 10^5^ cells/ml.

Splenic naive CD4^+^ CD44^low^ cells isolated *via* negative selection by magnetic bead purification (Miltenyi Biotec) from WT and Cbl-b^−/−^ mice were labeled with CFSE and stimulated with 2.5 µg/ml of plate-bound anti-CD3 ab, 1 µg/ml of soluble anti-CD28 ab in the presence of 10 µg/ml of plate-bound control Ig, or PD-L1 Ig for 48 h in 10% FCS complete RPMI 1640 in round-bottom 96-wells at 2.5 × 10^5^ cells/ml. These cells in both cultures were stained with Live/Dead Near IR (Live/Dead) (Molecular Probe), anti-CD8α, or anti-CD4 (Biolegend) and analyzed by flow cytometry using Becton Dickinson LSRII for CFSE dilution. Absolute numbers of cells in these cultures were quantified using MACSQuant Analyzer 10 (Miltenyi Biotec). For PD-1 expression, unstimulated splenocytes from WT and Cbl-b^−/−^ mice and CD8^+^ or CD4^+^ T cells stimulated in above conditions without Igs were surface-stained with Live/Dead, anti-CD8α or anti-CD4, anti-CD44, and anti-PD-1 (Biolegend) and analyzed by flow cytometry.

### *In Vitro* Suppression of T Cell IFN-γ Production by the Recombinant PD-L1 Ig

Splenic naïve CD8^+^ CD44^low^ T cells isolated *via* negative selection by magnetic bead purification (Miltenyi Biotec) from WT and Cbl-b^−/−^ mice were stimulated with 2 µg/ml of plate-bound anti-CD3 ab and 0.4 µg/ml of soluble anti-CD28 ab in the presence of 9–10 µg/ml of plate-bound control Ig or PD-L1 Ig for 72 h in 10% FCS complete RPMI 1640 in round-bottom 96-wells at 5 × 10^5^ cells/ml. Splenic naïve CD4^+^ CD44^low^ T cells isolated *via* negative selection by magnetic bead purification (Miltenyi Biotec) from WT and Cbl-b^−/−^ mice were stimulated with 2.5 µg/ml of plate-bound anti-CD3 ab and 1 µg/ml of soluble anti-CD28 ab in the presence of 10 µg/ml of plate-bound control Ig or PD-L1 Ig for 72 h in 10% FCS complete RPMI 1640 in round-bottom 96-wells at 2.5 × 10^5^ cells/ml. CD4^+^ T cells were then re-stimulated overnight with 2.5 µg/ml of plate-bound anti-CD3 ab and 1 µg/ml of soluble anti-CD28 ab without any Igs. Brefeldin A (5 µg/ml) (Sigma-Aldrich, St. Louis, MO, USA) was added to both cultures in the last 4 h. Cells were surface-stained with Live/Dead, anti-CD8α or anti-CD4 then fixed and permeabilized using BD Cytoperm/Cytofix kit (BD Biosciences, San Jose, CA, USA), intracellularly stained with anti-IFN-γ (BD Biosciences), and analyzed by flow cytometry.

### *In Vitro* Suppression of NK Cell IFN-γ Production by the Recombinant PD-L1 Ig

Splenic NK cells isolated *via* negative selection by magnetic bead purification (STEMCELL Technologies, Vancouver, BC, Canada) from WT and Cbl-b^−/−^ mice were cultured for 7 days in the presence of 1,000 U/ml recombinant human IL-2 (National Institutes of Health, Bethesda, MD) in 10% FCS complete RPMI 1640 (supplemented with 50 µM 2-mercaptoethanol, l-glutamine, penicillin/streptomycin, non-essential amino acids, and sodium pyruvate) in 24-wells at 2.5 × 10^5^ cells/ml. The purity of NK cells on day 7 was determined by surface-staining with Live/Dead, anti-CD3, anti-NK1.1, and anti-CD49b (eBioscience, San Diego, CA, USA) by flow cytometry, and the cell number per well for the stimulation was normalized based on the NK cell purity (CD3^−^ NK1.1^+^ CD49b^+^). These NK cells were stimulated with 15–20 µg/ml of plate-bound anti-NK1.1 ab (Biolegend) in the presence of 12.5–15 µg/ml of plate-bound control Ig or PD-L1 Ig and 5 µg/ml Brefeldin A for 5 h in round-bottom 96-wells at 5 × 10^5^ cells/ml. These stimulated NK cells and unstimulated NK cells (from 7-day IL-2 culture) were surface-stained with Live/Dead, fixed, and permeabilized as described above and intracellularly stained with anti-IFN-γ or anti-PD-1, and then analyzed by flow cytometry.

### Coculture of WT and Cbl-b^−/−^ T Cells

Splenic naïve CD8^+^ CD44^low^ T cells isolated *via* negative selection by magnetic bead purification (Miltenyi Biotec) from WT congenic mice (CD45.1^+^) and Cbl-b^−/−^ mice were labeled with CFSE and stimulated with 2 µg/ml of plate-bound anti-CD3 ab, 0.4 µg/ml of soluble anti-CD28 ab in the presence of 9 µg/ml of plate-bound control Ig or PD-L1 Ig for 72 h in 10% FCS complete RPMI 1640 in round-bottom 96-well plates at 5 × 10^5^ cells/ml. Single cultures were set up in which WT congenic naïve CD8^+^ T cells and Cbl-b^−/−^ CD8^+^ T cells were stimulated separately. Cocultures were set up in which these two naïve CD8^+^ T cell populations were cultured and stimulated together. Splenic naïve CD4^+^ CD44^low^ T cells isolated *via* negative selection by magnetic bead purification (Miltenyi Biotec) from WT congenic mice and Cbl-b^−/−^ mice were labeled with CFSE and stimulated with 2.5 µg/ml of plate-bound anti-CD3 ab, 1 µg/ml of soluble anti-CD28 ab in the presence of 15 µg/ml of plate-bound control Ig, or PD-L1 Ig for 48 h in 10% FCS complete RPMI 1640 in round-bottom 96-well plates at 2.5 × 10^5^ cells/ml. Single cultures were set up in which WT congenic naïve CD4^+^ T cells and Cbl-b^−/−^ naïve CD4^+^ T cells were stimulated separately. Cocultures were set up in which these two naïve CD4^+^ T cell populations were cultured and stimulated together. Cells in these cultures were stained with Live/Dead, anti-CD8α or anti-CD4, anti-CD45.1, and anti-CD45.2 (Biolegend) and analyzed by flow cytometry using Becton Dickinson LSRII for CFSE dilution.

### Liver Metastasis Model of B16 Melanoma and Anti-PD-1 Antibody Treatment

B16 (B16F10) cells were grown to approximately 75% confluency in 10% FCS complete RPMI 1640 (supplemented with l-glutamine, penicillin/streptomycin, non-essential amino acids, and sodium pyruvate). 1 × 10^6^ B16 (B16F10) cells were intra-splenically injected into WT and Cbl-b^−/−^ mice. For the experiment with anti-PD-1 ab treatment, only WT mice were administered 100 μg/mouse of either anti-PD-1 monoclonal ab (BioXCell, West Lebanon, NH, USA) or isotype control (BioXCell) intraperitoneally on the day of tumor injection and every other day. On days 7–9, the mice were perfused with saline, and the liver and spleen were harvested from each mouse. The images of each spleen and the front and the back side of each liver tissue were taken using Panasonic Lumix DMC-FS7 camera (Panasonic, Osaka, Japan). Using these images, the percentages of tumor foci area in total liver area were quantified for each mouse using ImageJ software (National Institutes of Health).

### Statistical Analysis

Data were analyzed by unpaired two-tailed Student’s *t-*test or one-way ANOVA using GraphPad Prism Version 6.0 (GraphPad Software, La Jolla CA, USA). Statistical significance was accepted at *p* < 0.05.

## Results

### Cbl-b^−/−^ T Cells Are Resistant to PD-L1 Ig-Mediated Suppression of Proliferation

**Figure 1:**
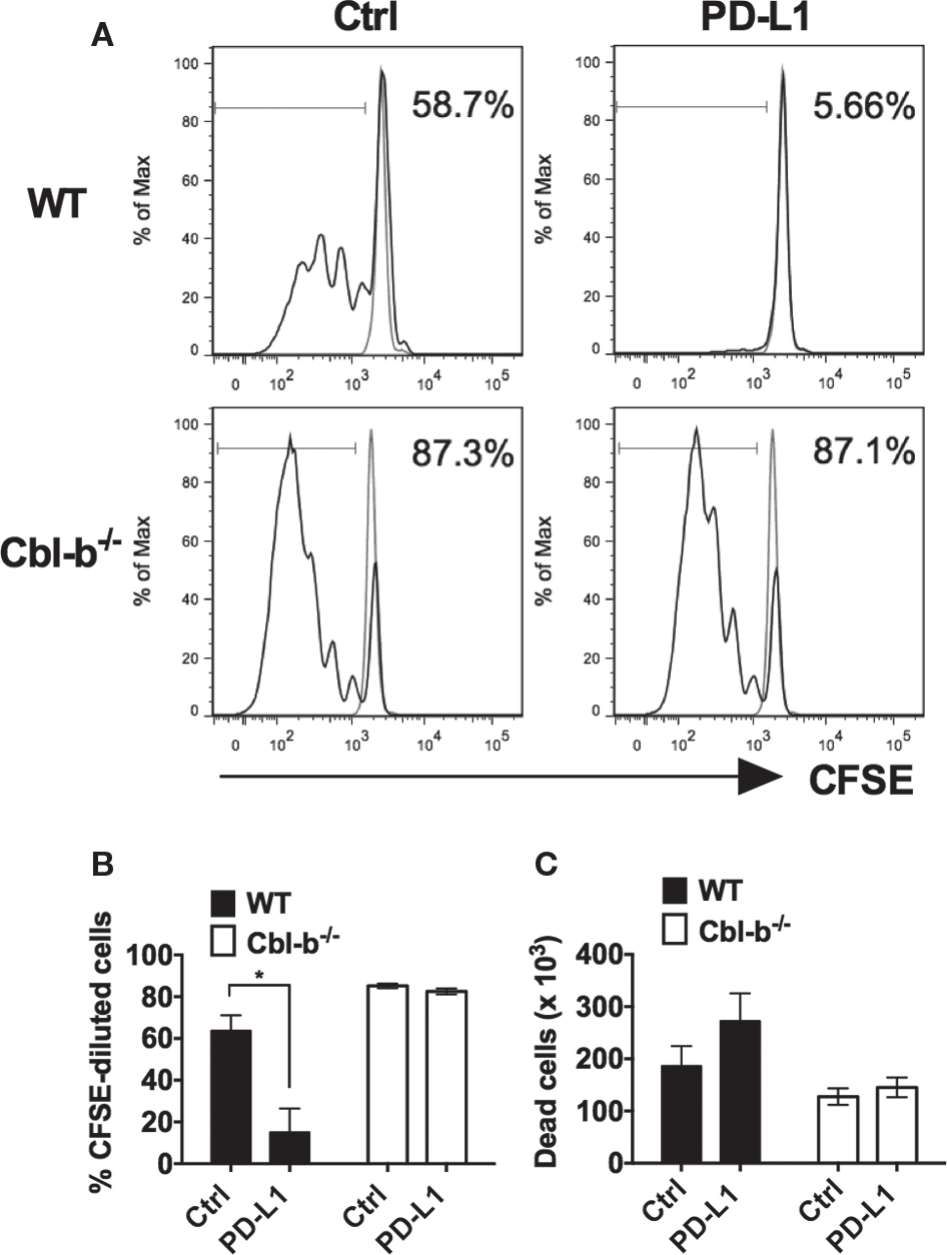
**Cbl-b^−/−^ CD8^+^ T cells are resistant to PD-L1 fusion protein (PD-L1 Ig)-mediated suppression of proliferation**. Purified splenic naive Cbl-b^−/−^ and WT CD8^+^ T cells were CFSE-labeled and stimulated for 3 days with plate-bound anti-CD3 ab and soluble anti-CD28 ab in the presence of either plate-bound control Ig or PD-L1 Ig. After 3 days, the percentage of CFSE-diluted cells was assayed by flow cytometry. **(A)** Representative FACS plots showing the percentages of CFSE-diluted cells in cultures of CD8^+^ T cells stimulated in the presence of control Ig or PD-L1 Ig (gated on live single CD8^+^ cells). Unstimulated cells are shown in gray. **(B)** Percentages of CFSE-diluted cells in cultures of CD8^+^ T cells stimulated in the presence of control Ig or PD-L1 Ig. **(C)** Number of dead cells in cultures of CD8^+^ T cells stimulated in the presence of control Ig or PD-L1 Ig. *n* = 3; Mean ± SEM depicted. Student’s *t*-test: *p < 0.05.

It has previously been demonstrated that *in vitro* T cell proliferation and cytokine production can be suppressed by the immobilized recombinant PD-L1 Ig (16, 24–26). To begin to test the sensitivity of Cbl-b^−/−^ T cells to PD-L1-mediated suppression, we tested the ability of plate-bound PD-L1 Ig to suppress the *in vitro* proliferation of WT and Cbl-b^−/−^ CD8^+^ T cells. Mouse splenic naïve CD44^low^ CD8^+^ T cells were purified from WT and Cbl-b^−/−^ mice and labeled with CFSE. These populations were then stimulated *in vitro* with plate-bound anti-CD3 ab and soluble anti-CD28 ab in the presence of either plate-bound PD-L1 Ig or plate-bound control human IgG_1_ (control Ig). After 3 days, cells were assayed for dilution of CFSE by flow cytometry. We found that TCR-stimulated *in vitro* proliferative responses of naïve WT CD8^+^ T cells were significantly suppressed by PD-L1 Ig (mean % CFSE-diluted cells: control Ig: 63.7%; PD-L1 Ig: 15.03%; *p* = 0.0234) (Figures 1A,B). In contrast, the proliferation of naïve Cbl-b^−/−^ CD8^+^ T cells was not suppressed by PD-L1 Ig (mean % CFSE-diluted cells: control Ig: 85.2%, mean PD-L1 Ig: 82.5%; *p* = 0.204) (Figures 1A,B). Thus, the mean percent suppression of CFSE-diluted WT CD8^+^ T cells by PD-L1 Ig was 79.6%, and that of CFSE-diluted Cbl-b^−/−^ CD8^+^ T cells was 3.13% (*p* = 0.0057) (Figure 1B). These differences in percent PD-L1 Ig-mediated suppression of CD8^+^ T cells were also reflected in absolute numbers of CFSE-diluted cells (data not shown).

To determine if the difference in PD-L1 Ig-mediated suppression was associated with differences in cell death, we compared the number of dead cells present in the control Ig wells with the number of dead cells in the PD-L1 Ig wells at the termination of the cultures. We found that PD-L1 Ig-mediated suppression of WT CD8^+^ T cells was associated with an increase in dead cells by 46%. In contrast, PD-L1 Ig-mediated suppression of Cbl-b^−/−^ CD8^+^ T cells was associated with an increase in dead cells by 13.7% (Figure 1C). Overall, our results suggest a resistance of Cbl-b^−/−^ CD8^+^ T cells to PD-L1 Ig-mediated suppression of proliferation.

**Figure 2:**
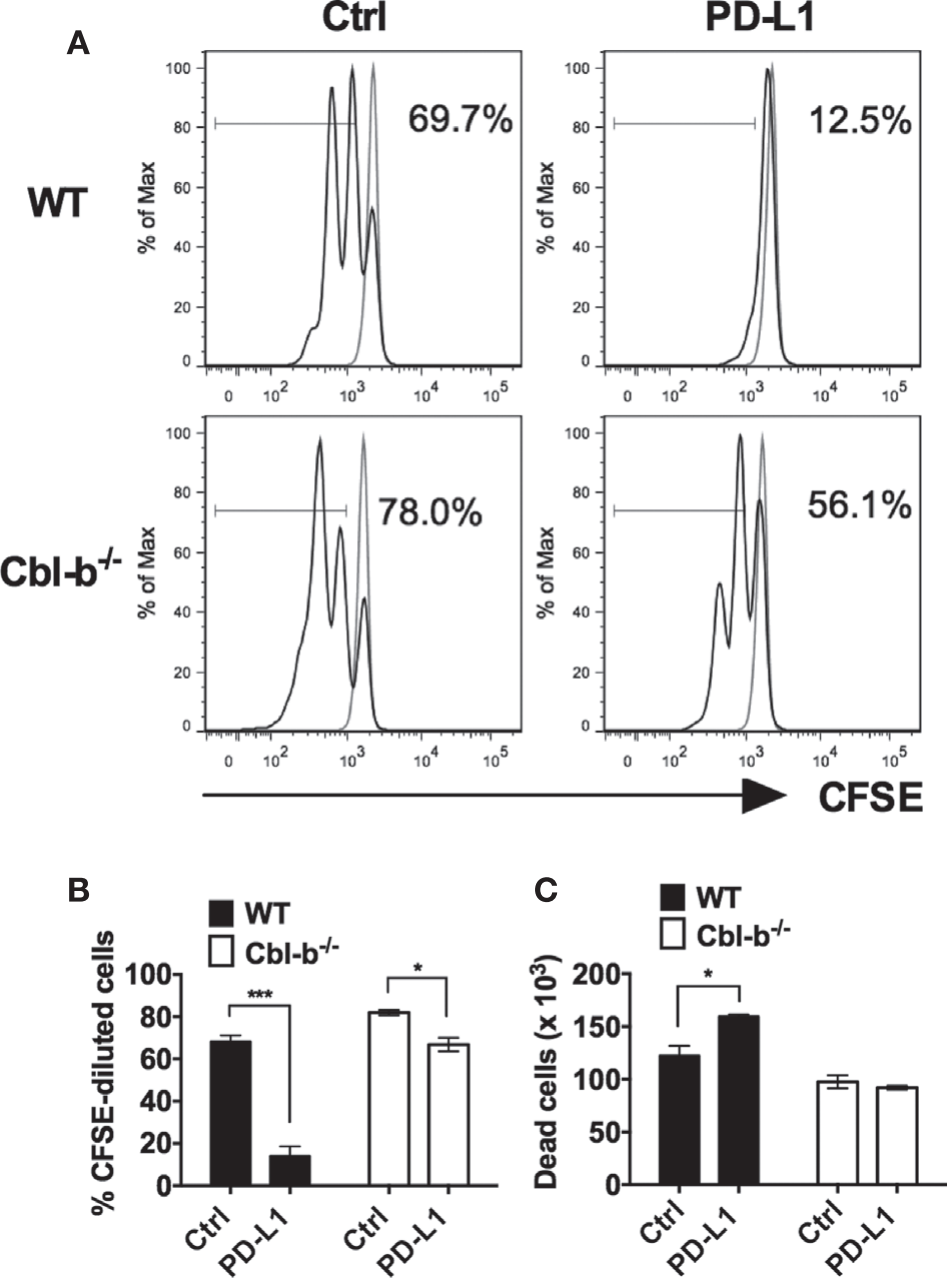
**Cbl-b^−/−^ CD4^+^ T cells are resistant to PD-L1 fusion protein (PD-L1 Ig)-mediated suppression of proliferation**. Purified splenic naïve Cbl-b^−/−^ and WT CD4^+^ T cells were CFSE-labeled and stimulated for 2 days with plate-bound anti-CD3 ab and soluble anti-CD28 ab in the presence of either plate-bound control Ig or PD-L1 Ig. After 2 days, the percentage of CFSE-diluted cells was assayed by flow cytometry. **(A)** Representative FACS plots showing the percentages of CFSE-diluted cells in cultures of CD4^+^ T cells stimulated in the presence of control Ig or PD-L1 Ig (gated on live single CD4^+^ cells). Unstimulated cells are shown in gray. **(B)** Percentages of CFSE-diluted cells in cultures of CD4^+^ T cells stimulated in the presence of control Ig or PD-L1 Ig. **(C)** Numbers of dead cells in cultures of CD4^+^ T cells stimulated in the presence of control Ig or PD-L1 Ig. *n* = 3; Mean ± SEM depicted. Student’s *t*-test: *p < 0.05, ***p < 0.001.

Next, we asked whether Cbl-b^−/−^ CD4^+^ T cells were also resistant to PD-L1 Ig-mediated suppression of *in vitro* proliferation. Mouse splenic naïve CD44^low^ CD4^+^ T cells isolated from WT and Cbl-b^−/−^ mice were labeled with CFSE and subjected to the similar stimulation cultures described above for CD8^+^ T cells. After 2 days, cells were assayed for dilution of CFSE by flow cytometry. Similar to CD8^+^ T cells, the *in vitro* proliferative responses of WT CD4^+^ T cells were significantly inhibited by PD-L1 Ig (mean % CFSE-diluted cells: control Ig: 68.4%; PD-L1 Ig: 14.1%; *p* = 0.0005) (Figures 2A,B). In contrast, the proliferative responses of Cbl-b^−/−^ CD4^+^ T cells were significantly less suppressed in the presence of PD-L1 Ig (mean % CFSE-diluted cells: control Ig: 82.0%; PD-L1 Ig: 66.9%; *p* = 0.0114) (Figures 2A,B). While a small and statistically significant degree of PD-L1 Ig-mediated suppression was noted in Cbl-b^−/−^ CD4^+^ T cells (mean percent suppression of Cbl-b^−/−^ CD4^+^ T cells: 18.4%), this level of suppression was markedly less than that seen in WT CD4^+^ T cells (mean percent suppression: 79.8%) (Figure 2B). These differences in percent PD-L1 Ig-mediated suppression of CD4^+^ T cells were also reflected in absolute numbers of CFSE-diluted cells (data not shown).

To determine if differences in PD-L1-mediated suppression between naïve WT versus Cbl-b^−/−^ CD4^+^ cells were associated with differences in cell death, we compared the number of dead cells present in the control Ig wells with the number of dead cells in PD-L1 Ig wells at the termination of cultures. We found that PD-L1-mediated suppression of WT CD4^+^ T cells was associated with an increase in dead cells by 30%. In contrast, PD-L1-mediated suppression of Cbl-b^−/−^ CD4^+^ T cells was not associated with a significant change in the number of dead cells (Figure 2C). Overall, as with Cbl-b^−/−^ CD8^+^ T cells, our results suggest a resistance of Cbl-b^−/−^ CD4^+^ T cells to PD-L1 Ig-mediated suppression of proliferation.

### The Expression of PD-1 Is Not Decreased in Cbl-b^−/−^ CD8^+^ and CD4^+^ T Cells

**Figure 3:**
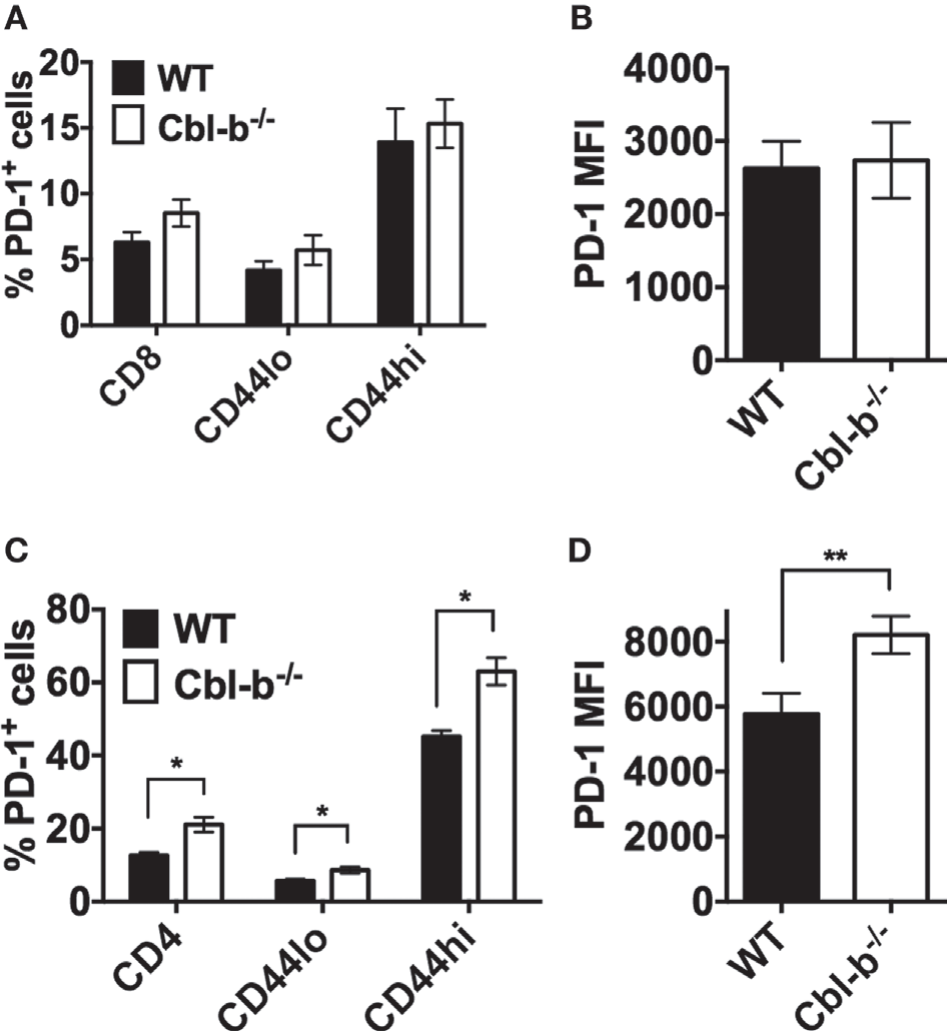
**The expression of PD-1 is not decreased in Cbl-b^−/−^ CD8^+^ and CD4^+^ T cells**. For *ex vivo* studies, splenocytes were derived from Cbl-b^−/−^ and WT mice and labeled with anti-CD8, anti-CD4, anti-PD-1, and anti-CD44 abs, immediately *ex vivo*. Alternatively, purified CD4^+^ and CD8^+^ T cells were labeled with anti-PD-1 ab after 2–3 days in culture with plate-bound anti-CD3 ab and soluble anti-CD28 ab. These cells were subsequently assayed by flow cytometry. **(A)** Frequencies of total CD8^+^, or CD8^+^ CD44^low^, or CD8^+^ CD44^high^ T cells that were PD-1^+^ in unstimulated splenocytes (gated on live, single, CD8^+^, CD44^low^, or CD44^hi^ cells). **(B)** MFI of PD-1 expression in *in vitro* stimulated CD8^+^ T cells (gated on live, single, CD8^+^ cells). **(C)** Frequencies of total CD4^+^, or CD4^+^ CD44^low^, or CD4^+^ CD44^high^ T cells that were PD-1^+^ in unstimulated splenocytes (gated on live, single, CD4^+^, CD44^low^, or CD44^hi^ cells). **(D)** MFI of PD-1 expression in *in vitro* stimulated CD4^+^ T cells (gated on live, single, CD4^+^ cells). *n* = 3; Mean ± SEM depicted. Student’s *t*-test: *p < 0.05, **p < 0.01.

To determine whether the resistance of Cbl-b^−/−^ T cells to PD-L1 Ig-mediated suppression is a result of decreased PD-1 expression, we compared the expression of PD-1 in WT versus Cbl-b^−/−^ CD8^+^ and CD4^+^ T cells before and after *in vitro* TCR stimulation. In CD8^+^ T cells, the frequency of PD-1^+^ cells was comparable between unstimulated WT and Cbl-b^−/−^ CD8^+^ T cells, and this was true for total CD8^+^ T cells, CD44^low^, and CD44^high^ CD8^+^ populations (Figure 3A). After 3 days of *in vitro* stimulation, approximately 95% of CD8^+^ T cells were positive for PD-1 expression. Therefore, we examined the MFI of PD-1 expression in these activated cells and found that the PD-1 MFI was comparable between Cbl-b^−/−^ and WT CD8^+^ T cells (Figure 3B). Unstimulated Cbl-b^−/−^ CD4^+^ T cells, when compared to unstimulated WT CD4^+^ T cells, demonstrated an increased frequency of PD-1^+^ cells. This was true for total CD4^+^ T cells, CD44^low^ and CD44^high^ CD4^+^ populations (Figure 3C). After 2 days of *in vitro* stimulation, the PD-1 MFI was significantly higher in Cbl-b^−/−^ CD4^+^ T cells compared to WT cells (Figure 3D). These results suggest that the resistance of Cbl-b^−/−^ T cells to PD-L1 Ig-mediated suppression is not a result of decreased PD-1 expression.

### PD-L1 Ig-Mediated Suppression of IFN-γ Is Significantly Greater for WT than for Cbl-b^−/−^ T Cells

We next asked whether Cbl-b^−/−^ T cells were resistant not only to PD-L1 Ig-mediated suppression of proliferation but also resistant to suppression of IFN-γ production *in vitro*. Splenic naïve CD44^low^ CD8^+^ T cells isolated from WT and Cbl-b^−/−^ mice were stimulated with anti-CD3 and anti-CD28 abs in the presence of plate-bound PD-L1 Ig or control Ig. After 3 days of culture, these cells were stained for intracellular IFN-γ, and analyzed by flow cytometry.

**Figure 4:**
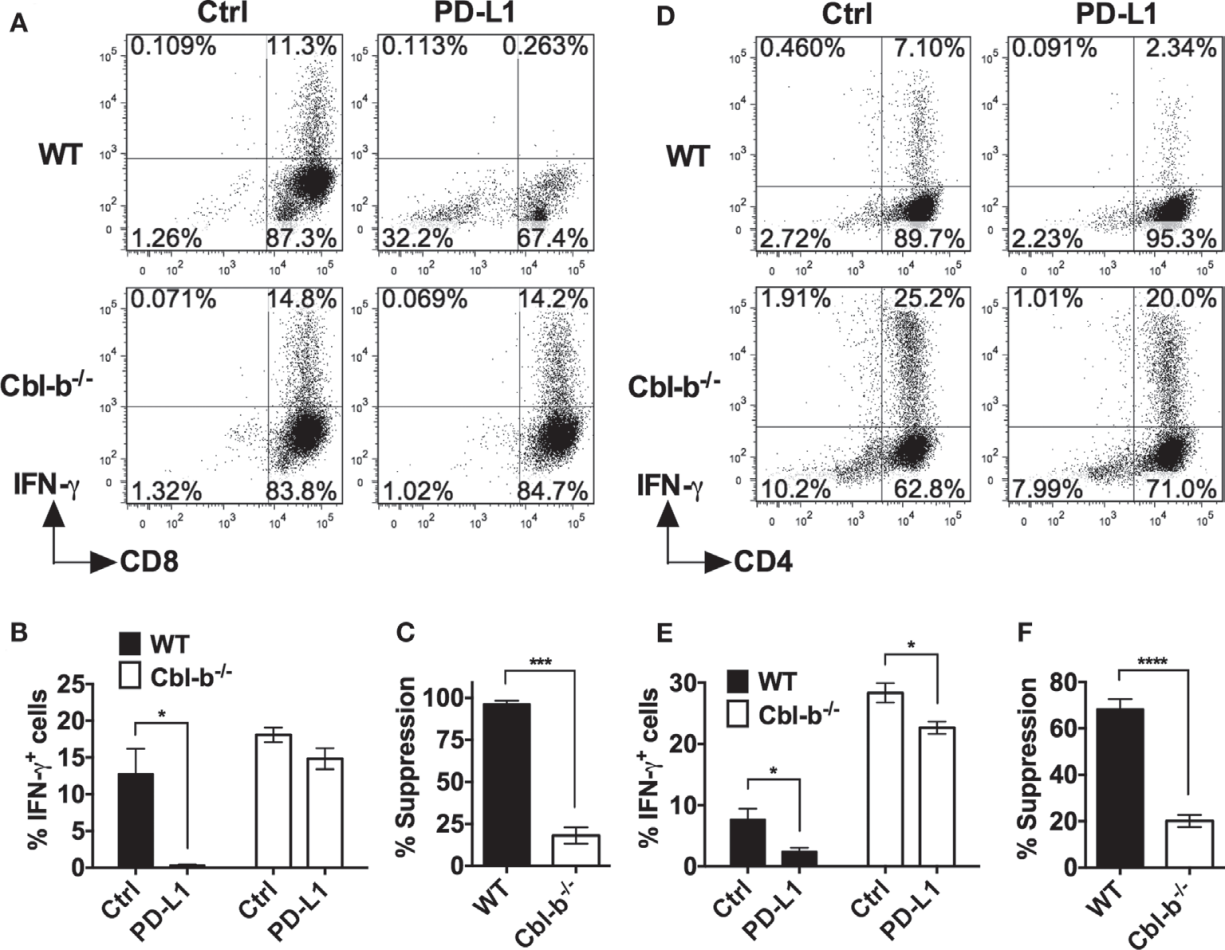
**PD-L1 fusion protein (PD-L1 Ig)-mediated suppression of IFN-γ is significantly greater for WT than for Cbl-b^−/−^ T cells**. Purified naïve splenic Cbl-b^−/−^ and WT CD8^+^ CD44^low^ and CD4^+^ CD44^low^ T cells were stimulated for 3 days with plate-bound anti-CD3 ab and soluble anti-CD28 ab in the presence of either plate-bound control Ig or PD-L1 Ig. For both CD8^+^ and CD4^+^ T cell cultures, brefeldin A was added in the last 4 h of culture, and cells were intracellularly stained with anti-IFN-γ ab and analyzed by flow cytometry. **(A)** Representative FACS plots showing the percentages of IFN-γ^+^ cells in cultures of CD8^+^ T cells stimulated in the presence of control Ig or PD-L1 Ig (gated on live single CD8^+^ cells). **(B)** Mean frequencies of IFN-γ-producing CD8^+^ T cells stimulated in the presence of either control Ig or PD-L1 Ig. **(C)** Mean percentage suppression of CD8^+^ T cell IFN-γ production by PD-L1 Ig as shown in panel **(B)**. **(D)** Representative FACS plots showing the percentages of IFN-γ^+^ cells in cultures of CD4^+^ T cells stimulated in the presence of control Ig or PD-L1 Ig (gated on live single CD4^+^ cells). **(E)** Mean frequencies of IFN-γ-producing CD4^+^ T cells stimulated in the presence of control Ig or PD-L1 Ig. **(F)** Mean percentage suppression of CD4^+^ T cell IFN-γ production by PD-L1 Ig as shown in panel **(E)**. Mean ± SEM depicted. *n* = 3 for panels **(B)** and **(C)**; *n* = 5–6 for panels **(E)** and **(F)**. Student’s *t*-test: *p < 0.05, ***p < 0.001, ****p < 0.0001.

The frequency of IFN-γ-producing WT CD8^+^ T cells was significantly suppressed by PD-L1 Ig (mean IFN-γ positive cells: control Ig: 12.8%; PD-L1 Ig: 0.3%; *p* = 0.0221) (Figures 4A,B). In contrast, the frequency of IFN-γ-producing Cbl-b^−/−^ CD8^+^ T cells was not statistically suppressed by PD-L1 Ig (mean IFN-γ positive cells: control Ig: 18.1%, mean PD-L1 Ig: 14.8%; *p* = 0.1374) (Figures 4A,B). Thus, the level of suppression was significantly lower in Cbl-b^−/−^ CD8^+^ T cells than that seen in WT CD8^+^ T cells (mean percent suppression of IFN-γ-producing cells: WT CD8^+^: 96.3%; Cbl-b^−/−^ CD8^+^: 18.1%; *p* = 0.0001) (Figure 4C).

We next stimulated WT and Cbl-b^−/−^ splenic naïve CD44^low^ CD4^+^ T cells with anti-CD3 and anti-CD28 abs in the presence of plate-bound PD-L1 Ig or control Ig. As with WT CD8^+^ T cells, the frequency of IFN-γ-producing WT CD4^+^ T cells was significantly suppressed by PD-L1 Ig (mean IFN-γ positive cells: control Ig: 7.7%; PD-L1 Ig: 2.4%; *p* = 0.0177) (Figures 4D,E). The frequency of IFN-γ-producing Cbl-b^−/−^ CD4^+^ T cells was also suppressed by PD-L1 Ig (mean IFN-γ positive cells: control Ig: 28.4%; PD-L1 Ig: 22.6%; *p* = 0.0146) (Figures 4D,E), but the level of suppression was significantly less than that seen with WT CD4^+^ T cells (mean percent suppression of IFN-γ-producing cells: WT CD4^+^: 68.3%; Cbl-b^−/−^ CD4^+^: 20.1%; *p* < 0.0001) (Figure 4F). Thus, consistent with the proliferation results, Cbl-b^−/−^ CD8^+^ and CD4^+^ T cell IFN-γ production is significantly less suppressed by PD-L1 Ig than is WT CD8^+^ and CD4^+^ T cell IFN-γ production.

### Cbl-b^−/−^ NK Cells Are Resistant to PD-L1-Mediated Suppression of IFN-γ Production

The role of NK cells has been documented in antitumor immunity in mice and humans, and these cells have been shown to contribute to the robust antitumor immunity of Cbl-b^−/−^ mice (14). Similar to T cells, NK cells can also express PD-1 and the engagement of PD-1 on NK cells inhibits their activation and cytotoxic function (27). We, next, asked whether Cbl-b^−/−^ NK cells were also resistant to PD-L1/PD-1 suppression.

**Figure 5:**
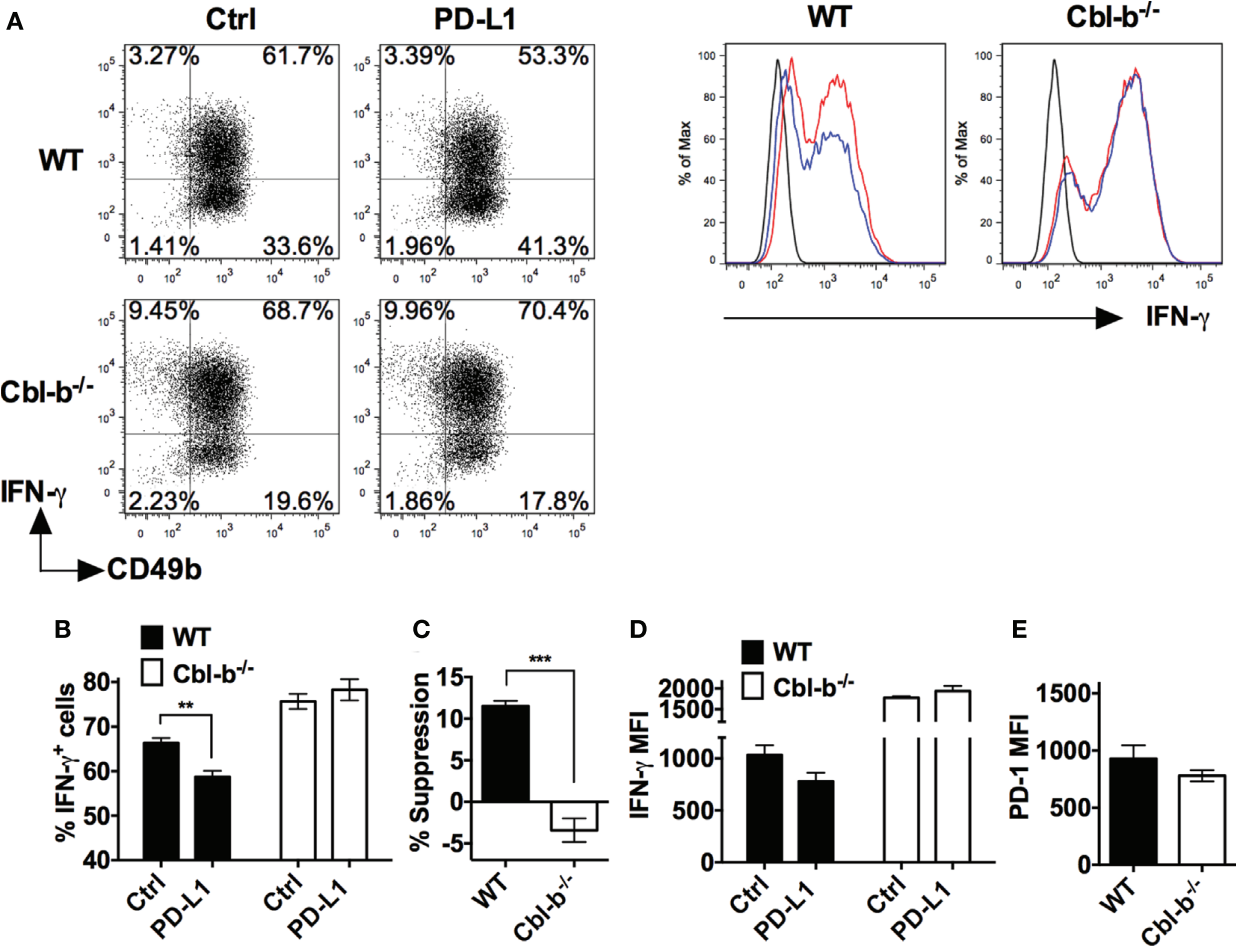
**Cbl-b^−/−^ NK cells are resistant to PD-L1 fusion protein (PD-L1 Ig)-mediated suppression of IFN-γ production**. Splenic NK cells were isolated from Cbl-b^−/−^ and WT mice and cultured for 7 days in the presence of IL-2. These NK cells were harvested from the 7-day cultures and stimulated with plate-bound anti-NK1.1 ab in the presence of either plate-bound control Ig or plate-bound PD-L1 Ig and brefeldin A for 5 h. The NK cells were then intracellularly stained with anti-IFN-γ ab and analyzed by flow cytometry. Additionally, unstimulated NK cells were stained with anti-PD-1 ab after the 7-day IL-2 culture and analyzed by flow cytometry. **(A)** Representative dot blots and corresponding histograms on 7-day-cultured NK cells stained for intracellular IFN-γ. Cells were gated on live single WT or Cbl-b^−/−^ CD3^−^ (dot blots) or CD3^−^ CD49b^+^ cells (histograms) that had been stimulated in the presence of control Ig or PD-L1 Ig. In histograms: black line represents isotype staining, red line represents control Ig, and blue line represents PD-L1 Ig. **(B)** Mean frequencies of IFN-γ-producing WT or Cbl-b^−/−^ NK cells stimulated in the presence of control Ig or PD-L1 Ig (gated on live single CD3^−^ CD49b^+^ cells). **(C)** Mean percentage suppression of IFN-γ-producing NK cells after stimulation in the presence of PD-L1 Ig as shown in panel **(A)**. **(D)** Mean MFI of IFN-γ staining in 7-day cultured WT or Cbl-b^−/−^ NK cells stimulated in the presence of control Ig or PD-L1 Ig (gated on live single CD3^−^ CD49b^+^ cells). **(E)** Mean MFI by flow cytometry of PD-1 expression in unstimulated WT and Cbl-b^−/−^ NK cells that were harvested from the 7-day IL-2 culture (gated on live single CD3^−^ NK1.1^+^ CD49b^+^ cells). Mean ± SEM depicted. *n* = 3. Student’s *t*-test: **p < 0.01, ***p < 0.001.

WT and Cbl-b^−/−^ splenic NK cells were isolated and initially cultured in the presence of IL-2 as previously described (28). The mean purity or percentages of NK cells after 7 days of culture with IL-2 was WT = 97.57% and Cbl-b^−/−^ = 96.3% when gated on CD3^−^ NK1.1^+^ cells. After 7 days of culture, WT NK cells contained an average of 1.7% CD3^+^ T cells, and Cbl-b^−/−^ NK cells contained an average of 0.5% CD3^+^ T cells (data not shown). The NK cells were then stimulated *in vitro* with plate-bound anti-NK1.1 ab in the presence of either plate-bound PD-L1 Ig or control Ig and IFN-γ production measured by intracellular staining and flow cytometry. Additionally, the expression of PD-1 on the unstimulated NK cells was examined by flow cytometry. The frequency of IFN-γ-producing WT NK cells after stimulation with plate-bound anti-NK1.1 ab was significantly suppressed by PD-L1 Ig (*p* = 0.0022) (Figures 5A,B). In contrast, the frequency of IFN-γ-producing Cbl-b^−/−^ NK cells was not suppressed by PD-L1 Ig (Figures 5A,B). Overall, the mean percentage of PD-L1 Ig-mediated suppression of IFN-γ-producing WT NK cells was 11.5%, while the mean percent suppression of IFN-γ-producing Cbl-b^−/−^ NK cells was −3.4% (*p* = 0.0006) (Figure 5C). Although it did not reach statistical significance, PD-L1 Ig reduced the IFN-γ expression of WT NK cells based on the MFI, while that of Cbl-b^−/−^ NK cells was not reduced by PD-L1 Ig (Figure 5D). Finally, PD-1 expression by Cbl-b^−/−^ NK cells was slightly, but not statistically, lower than PD-1 expression on WT NK cells (*p* = 0.304) (Figure 5E). These results suggest that, in addition to Cbl-b^−/−^ T cells, Cbl-b^−/−^ NK cells are also significantly less sensitive to suppression by PD-L1 Ig.

### Does Coculturing with Cbl-b^−/−^ T Cells Result in Bystander WT T Cell Resistance to PD-L1-Mediated Suppression?

To begin to investigate the potential functional relevance of Cbl-b^−/−^’s resistance to PD-L1 suppression, we focused on reports documenting that enhanced levels of certain common γ chain-signaling cytokines may have a role in overcoming PD-L1-mediated suppression (29). It has been documented that activated Cbl-b^−/−^ T cells secrete increased levels of IL-2 compared to WT T cells (1, 2). Therefore, we next explored whether this might result in the induction of bystander PD-L1 resistance in WT T cells. We cultured naïve Cbl-b^−/−^ T cells or naïve WT T cells either separately or together and then asked whether factors secreted by activated Cbl-b^−/−^ T cells would induce PD-L1 resistance in bystander (cocultured) WT T cells.

**Figure 6:**
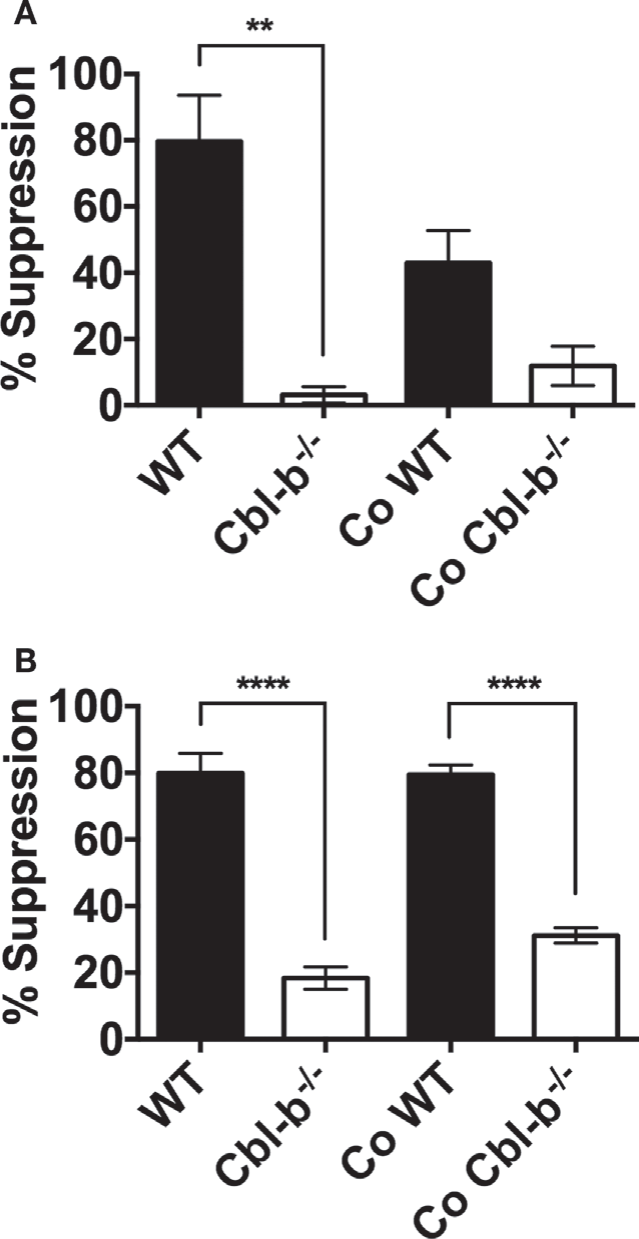
**PD-L1 fusion protein (PD-L1 Ig)-suppression in cocultures of Cbl-b^−/−^ and WT T cells**. Naïve CD8^+^ CD44^low^ T cells and naïve CD4^+^ CD44^low^ T cells were isolated from spleens of WT C57BL/6, CD45.1^+^ congenic mice, and Cbl-b^−/−^ mice (CD45.2^+^). WT and Cbl-b^−/−^ T cells were CFSE-labeled and stimulated for 3 (CD8^+^) or 2 (CD4^+^) days either separately (WT and Cbl-b^−/−^ T cells in separate cultures) or together (“Co” = WT and Cbl-b^−/−^ T cells cultured together) with plate-bound anti-CD3 ab and soluble anti-CD28 ab in the presence of either plate-bound control Ig or PD-L1 Ig. At the end of culture, the percentage of CFSE-diluted cells was assayed by flow cytometry. **(A)** Mean percentage suppression by PD-L1 Ig of single and cocultures of naïve CD8^+^ CD44^low^ T cells stimulated in the presence of control Ig or PD-L1 Ig. Co-WT indicates % suppression of WT CD8^+^ T cells by PD-L1 in the coculture of WT and Cbl-b^−/−^ CD8^+^ T cells. Co-Cbl-b^−/−^ indicates % suppression of Cbl-b^−/−^ CD8^+^ T cells by PD-L1 Ig in the same coculture. **(B)** Mean percentage suppression by PD-L1 Ig of single and cocultures of naïve CD4^+^ CD44^low^ T cells stimulated in the presence of control Ig or PD-L1 Ig. Co-WT indicates % suppression of WT CD4^+^ T cells by PD-L1 in the coculture of WT and Cbl-b^−/−^ CD4^+^ T cells. Co-Cbl-b^−/−^ indicates % suppression of Cbl-b^−/−^ CD4^+^ T cells by PD-L1 Ig in the same coculture. *n* = 3; Mean ± SEM depicted. One-way ANOVA: **p < 0.01, ****p < 0.0001.

In the CD8^+^ T cell cultures, there was a clear, though not statistically significant (*p* = 0.08) effect of naive Cbl-b^−/−^ CD8^+^ T cells in diminishing the PD-L1 Ig-mediated suppression of bystander naïve WT CD8^+^ T cells (Figure 6A). The mean PD-L1 Ig-mediated suppression of WT CD8^+^ T cells in single culture was 79.6%, and this suppression decreased to a mean of 42.9% when WT CD8^+^ T cells were cocultured with Cbl-b^−/−^ CD8^+^ T cells (Figure 6A). These results suggest that Cbl-b^−/−^ CD8^+^ T cells can induce a partial resistance of bystander WT CD8^+^ T cells to PD-L1 Ig-mediated suppression. In contrast, the level of PD-L1 Ig-mediated suppression of WT CD4^+^ T cells remained unchanged, regardless of whether WT CD4^+^ T cells were cultured alone or cocultured together with Cbl-b^−/−^ CD4^+^ T cells (Figure 6B). In terms of potential functional relevance, these results suggest that the adoptive transfer of CD8^+^ T cells in which the expression of the *CBLB* gene has been silenced may be accompanied by a loss of sensitivity to PD-L1-mediated suppression among bystander endogenous T cells.

### Cbl-b^−/−^ Mice Develop Significantly Fewer Metastases in a PD-L1/PD-1-Dependent Model of B16 Melanoma Liver Metastasis

As Cbl-b^−/−^ T cells were resistant to PD-L1 Ig-mediated regulation *in vitro*, we sought to test this in an *in vivo* B16 melanoma tumor model in which inhibiting the PD-L1/PD-1 pathway has been shown to prevent the outgrowth of liver metastases (30). In this model, splenic injection of B16 melanoma in WT mice results in significant metastatic foci in the liver. However, if anti-PD-1 antibody is administered, liver metastases are significantly reduced (30). In addition, Iwai et al. documented that PD-1^−/−^ mice develop significantly fewer liver metastases compared to WT mice in this model (30). Thus, PD-L1/PD-1-mediated immune regulation appears to normally allow spread of the melanoma to the liver, while inhibiting or deleting this normal PD-1/PD-L1-mediated immune regulation prevents the spread of the tumor to the liver. This model allows us to ask whether Cbl-b^−/−^ mice can eliminate the B16 liver metastases on their own, i.e., in the absence of anti-PD-1 antibody, and thus to assess the functional relevance of the *in vitro* resistance of Cbl-b^−/−^ T cells to PD-L1/PD-1 in an *in vivo* tumor setting.

In using this model, Cbl-b^−/−^ mice could potentially eliminate liver metastases on the basis of their resistance to TGF-β or Tregs (7–11). However, if Cbl-b^−/−^ mice were *unable* to eliminate the liver metastases in the absence of anti-PD-1 antibody, this would suggest that Cbl-b^−/−^ mice are *not* resistant to PD-L1/PD-1 suppression *in vivo*. In contrast, demonstrating that Cbl-b^−/−^ mice can indeed eliminate the liver metastases in the absence of anti-PD-1 antibody would minimally suggest a lack of dominant sensitivity to PD-L1/PD-1 suppression *in vivo* and thus be consistent with our *in vitro* findings.

**Figure 7:**
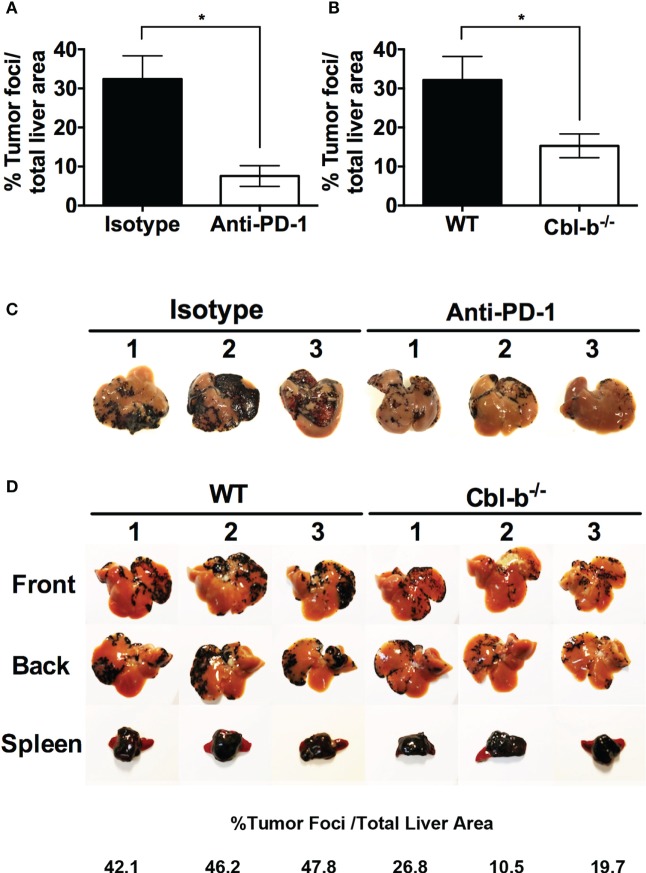
**Cbl-b^−/−^ mice develop significantly fewer liver metastases in a PD-L1/PD-1-dependent model of B16 melanoma liver metastasis**. **(A)** WT mice received an intra-splenic injection of B16 melanoma cells and then were treated with either 100 µg of isotype control ab or anti-PD-1 ab i.p. on the day of tumor injection and every other day until the mice were sacrificed. The mice were sacrificed on day 8, perfused with saline, the liver and the spleen harvested, and the percentages of tumor foci area/total liver area were quantified using ImageJ software. **(B)** WT and Cbl-b^−/−^ mice received an intra-splenic injection of B16 melanoma cells as in panel **(A)**, but no isotype or anti-PD-1 ab was given. The mice were sacrificed on day 7–9, perfused with saline, the liver and the spleen harvested, and the percentages of tumor foci area/total liver area were quantified using ImageJ software. **(C)** Images of the livers from the experiment shown in panel **(A)**. **(D)** Representative images of liver and spleen from panel **(B)** with percentages of tumor foci area/total liver area for the livers presented. Mean ± SEM depicted. *n* = 3 for panel **(A)**, *n* = 12–14 for panel **(B)**. Student’s *t*-test: *p = 0.019 for panel **(A)**; *p = 0.026 for panel **(B)**.

We first confirmed using WT mice that the model was sensitive to anti-PD-1 antibody treatment. WT mice were injected intra-splenically with B16 melanoma and treated with either anti-PD-1 ab or an isotype control. As reported by Iwai et al (30), WT mice treated with anti-PD-1 ab developed significantly fewer liver metastases than mice treated with isotype control (Figures 7A,C). No difference in the “primary” spleen tumor masses was noted between mice treated with anti-PD-1 ab or isotype (data not shown). Next, WT and Cbl-b^−/−^ mice were injected intra-splenically with B16 melanoma and the presence of liver metastases was analyzed 7–9 days after the tumor injection. No anti-PD-1 ab or isotype treatment was given to these mice. Strikingly, we found that Cbl-b^−/−^ mice developed significantly fewer liver metastases compared to WT mice (Figures 7B,D). In contrast, the primary spleen tumor masses were comparable between WT and Cbl-b^−/−^ mice in all of the experiments (Figure 7D), suggesting that Cbl-b^−/−^ mice are not generally capable of eliminating the B16 melanoma based on their resistance to TGF-β or Tregs (7–11). Our finding that Cbl-b^−/−^ mice develop significantly fewer liver metastases compared to WT mice without the administration of anti-PD-1 antibody minimally suggests a lack of dominant sensitivity to PD-L1/PD-1 suppression *in vivo* and thus is consistent with our *in vitro* findings of resistance to PD-L1/PD-1-mediated suppression.

## Discussion

Casitas B-lineage lymphoma-b is an E3 ubiquitin ligase that negatively regulates T cell activation (1–4). Cbl-b^−/−^ mice demonstrate spontaneous autoimmunity and robust antitumor immunity.

Recently, enhancement of antifungal immunity has also been documented in Cbl-b^−/−^ mice (31–33). The enhanced antitumor responses of Cbl-b^−/−^ mice have been documented in tumor models of EL4 (11), EG.7 (10), TC-1 (4, 9), UVB-induced skin tumor (9), and ATM-deficiency-induced T cell lymphoma (10). The T cell-intrinsic role of the enhanced antitumor immunity of Cbl-b^−/−^ mice has been demonstrated in adoptive T cell transfer experiments using Cbl-b^−/−^ or *Cblb*-silenced CD8^+^ T cells (9, 10, 34–36). In an attempt to elucidate the mechanisms underlying the robust antitumor activity of Cbl-b^−/−^ T cells, we and others have shown that Cbl-b^−/−^ T cells are resistant to suppression by Tregs (7–9) and TGF-β (10, 11). Because of the enhanced antitumor responses of Cbl-b^−/−^ mice, Cbl-b has become a focus for understanding mechanisms of antitumor immunity and a target for manipulation in antitumor immunotherapeutic approaches. In this regard, siRNA-mediated knockdown of *CBLB* is currently being investigated as a therapeutic approach to enhance antitumor responses in cancer patients (34, 36, 37). Similar to the findings in mouse Cbl-b^−/−^ CD8^+^ T cells, *CBLB*-silenced human CD8^+^ T cells demonstrate enhanced IFN-γ production (34, 36), reduced sensitivity to TGF-β (36), and independence from co-stimulation (36) and exogenous IL-2 in activation and proliferation (34). These findings in human *CBLB*-silenced T cells suggest their potential for mediating robust antitumor activity *in vivo* and underlie the high level of interest in utilizing *CBLB* silencing in treating human cancer patients.

PD-1 belongs to the CD28/B7 family of co-stimulatory molecules and is expressed on activated CD8^+^ and CD4^+^ T cells, NK and NKT cells, B cells, activated monocytes, and some dendritic cells (38). Triggering of PD-1 in T cells by its ligands PD-L1 and PD-L2 has been shown to inhibit activation and dampen subsequent proliferation and cytokine production (15, 16, 24–26). The expression of PD-L1 on tumors has been shown to increase the invasiveness of the tumor in mice and blockade of PD-L1/PD-1 interaction has been shown to halt tumor progression in mouse models (30, 39). Furthermore, the recent demonstration of the efficacy of the PD-L1/PD-1 blockade for the treatment of certain human cancers underscores the importance of inhibiting this pathway for effective antitumor immunity (17–19). Despite the documented ability of Cbl-b^−/−^ mice to mount enhanced antitumor responses, to date, there has been no investigation of the relationship of Cbl-b deficiency and the PD-L1/PD-1 pathway. In the present study, we now report a relationship with potential translational significance between Cbl-b deficiency and a resistance to PD-L1/PD-1-mediated immune regulation. This concept is supported by the following specific results in our study.

We find that *in vitro* proliferative responses of Cbl-b^−/−^ CD8^+^ and CD4^+^ T cells are significantly less suppressed by a recombinant PD-L1 Ig than are those of WT T cells.

In addition to the proliferative responses, *in vitro* IFN-γ production of Cbl-b^−/−^ CD8^+^ and CD4^+^ T cells is also significantly less suppressed by PD-L1 Ig compared to WT T cells. The PD-1/PD-L1 resistance of Cbl-b^−/−^ T cells is not a result of a difference in PD-1 expression in Cbl-b^−/−^ CD8^+^ and CD4^+^ T cells. Recently, Cbl-b^−/−^ mice have been reported to have NK cells with enhanced antitumor activity (14). This enhancement of antitumor activity in Cb-b^−/−^ NK cells was reported to be associated with their resistance to TAM (Tyro3, Axl, and Mer) receptor-mediated inhibition (14). Similar to T cells, NK cells can express PD-1, and the engagement of PD-1 on NK cells inhibits their activation and cytotoxic function (27). We asked whether the enhanced antitumor activity of Cbl-b^−/−^ NK cells might also be related to a resistance to PD-L1/PD-1-mediated suppression. We now find that in contrast to WT NK cells, Cbl-b^−/−^ NK cells are not suppressed in *in vitro* IFN-γ production by PD-L1. Thus, we have described an additional mechanism by which Cbl-b^−/−^ NK cells may be capable of mediating enhanced antitumor activity.

To begin to identify the potential functional relevance of resistance to PD-L1 Ig-mediated suppression in Cbl-b^−/−^ T cells, we asked whether Cbl-b^−/−^ T cells could induce resistance to PD-L1-mediated suppression in bystander WT T cells. Although not statistically significant, we find an induction of bystander resistance in naïve WT CD8^+^ T cells when cultured with naïve Cbl-b^−/−^ CD8^+^ T cells. There is presently a significant focus on developing approaches to silence *CBLB* in CD8^+^ T cells for subsequent use in enhancing antitumor immunity (34, 36, 40). Our coculture results suggest that the adoptive transfer of CD8^+^ T cells in which *CBLB* has been silenced may result in a significant enhancement of T cell antitumor responses through both PD-L1 resistance in the *CBLB*-silenced T cells and induction of bystander PD-L1 resistance in the endogenous T cell populations.

Finally, we examined the *in vivo* relevance of the resistance of Cbl-b^−/−^ T cells to PD-L1/PD-1 utilizing a previously established tumor model in which the spread of intra-splenically injected B16 melanoma requires functionally intact PD-L1/PD-1 regulation (30). The ideal system for testing the PD-1/Cbl-b interaction *in vivo* would require a model in which PD-L1/PD-1 suppression can be analyzed without other confounding suppressive influences. Current models only allow us to interrogate systems in which PD-L1/PD-1 has been shown to play a crucial role, while not directly ruling out the influence of other pathways. As such, we chose a model system in which blockade of PD-1 is known to be effective in eradicating liver metastases.

Strikingly, we found that, in the absence of anti-PD-1 treatment, Cbl-b^−/−^ mice develop far fewer metastatic tumor foci in the liver compared to WT mice. It should be noted that simultaneously, we found that the “primary” spleen tumor masses were comparable between WT and Cbl-b^−/−^ mice. This suggests that Cbl-b^−/−^ mice are not broadly capable of eliminating the B16 melanoma in all clinical settings, for example as a result of their resistance to TGF-β or Tregs (7–11). Moreover, this apparent lack of relevance of PD-L1/PD-1 regulation in splenic tumor outgrowth is consistent with the finding of Iwai et al., who reported that when B16 melanoma is administered subcutaneously, PD-1^−/−^ mice demonstrate tumor growth which is comparable to that seen in WT mice (30, 39). In sum, our finding that Cbl-b^−/−^ mice develop significantly fewer liver metastases compared to WT mice suggests, minimally, a lack of dominant sensitivity of Cbl-b^−/−^ mice to PD-L1/PD-1 suppression *in vivo* and thus is consistent with our *in vitro* findings of resistance to PD-L1/PD-1-mediated suppression.

There are several potential mechanisms by which Cbl-b^−/−^ T cells may resist suppression by PD-L1/PD-1. Karwacz et al. have previously reported that PD-1/PD-L1 interaction following TCR/CD28 stimulation induces both the upregulation of Cbl-b and TCR down-modulation in CD8^+^ T cells (23). Cbl-b^−/−^ CD8^+^ and CD4^+^ T cells have previously been shown to be defective in TCR down-modulation during T cell priming (41, 42). Based on these findings, Karwacz et al. have proposed that during PD-L1/PD-1 signaling, Cbl-b is required for the TCR down-modulation leading to the inhibition of T cell responses (23). However, the requirement for Cbl-b in the PD-L1/PD-1-mediated inhibition of T cell responses was never directly demonstrated in their study through the use of Cbl-b^−/−^ T cells. Moreover, Karwacz et al. did not observe TCR down-modulation in response to PD-L1/PD-1 signaling in CD4^+^ T cells and only focused on CD8^+^ T cells in their study. However, as noted above, it has been reported that both Cbl-b^−/−^ CD8^+^ and CD4^+^ T cells are defective in TCR down-modulation in response to *in vitro* stimulation (41, 42). This suggests that the resistance to PD-L1-mediated suppression we now report for both Cbl-b^−/−^ CD8^+^ and CD4^+^ T cells may involve mechanisms other than a resistance to PD-L1-induced TCR down-modulation.

Perhaps related to this concept, it has been reported that PD-L1/PD-1 signaling may act downstream by interacting with inhibitory phosphatases, such as SHP-1 and SHP-2 (22, 25, 26). In this regard, Xiao et al. recently demonstrated that after TCR stimulation, SHP-1 dephosphorylates Cbl-b to prevent its degradation (43). Thus, in the context of both TCR and PD-1 signaling in T cells, SHP-1 may also act to prevent degradation of Cbl-b, allowing Cbl-b to optimally inhibit its targets such as PI3K through its E3 ubiquitin ligase activity. PI3K has been described as one of the proximal TCR signaling molecules affected by PD-L1/PD-1 inhibitory signals along with Zap70 and PKC-θ (20, 21). Furthermore, Xiao et al. also documented that CD28 co-stimulatory signals block the interaction between SHP-1 and Cbl-b, allowing the degradation of Cbl-b. CD28 co-stimulation has previously been described to allow both mouse and human T cells to overcome suppression by PD-L1/PD-1 (29, 44). The disrupted interaction between SHP-1 and Cbl-b may be one of the underlying mechanisms by which CD28 co-stimulation diminishes PD-L1/PD-1 inhibitory signals (44).

Finally, Cbl-b^−/−^ T cells demonstrate increased production of IL-2 (1, 2). Exogenous IL-2 and certain other γ chain-signaling cytokines have been demonstrated to enable mouse and human T cells to overcome PD-L1/PD-1 inhibitory signals (29, 44). Our coculture studies suggest that enhanced cytokine secretion by Cbl-b^−/−^ CD8^+^ T cells may induce resistance to PD-L1/PD-1-mediated suppression in bystander WT CD8^+^ T cells. This also suggests that enhanced cytokine secretion may be playing a mechanistic role, in an autocrine fashion, in the PD-L1/PD-1 resistance in Cbl-b^−/−^ CD8^+^ T cells. Our results indicate however, that both of these characteristics may not be the case for CD4^+^ T cells. At present it is unclear why bystander resistance is noted in CD8^+^ but not in CD4^+^ cocultures. Future studies in our laboratory will explore this dichotomy and further identify the specific mechanisms by which Cbl-b plays a role in mediating the PD-L1/PD-1 inhibitory signals.

In sum, we have documented for the first time that Cbl-b deficiency in mice results in functional resistance of T cells and NK cells to PD-L1/PD-1-mediated immune regulation. Cbl-b^−/−^ mice develop only a mild autoimmune phenotype consisting of anti-dsDNA antibodies and variably, some degree of multi-organ lymphocyte infiltration. These findings occur only after approximately 9 months of age. As in all treatments that involve checkpoint inhibition in human cancer therapy, the use of T cells in which Cbl-b is inactivated will require a close watch for the development of autoimmune diseases Nevertheless, our results suggest that targeting Cbl-b in cancer immunotherapy offers the opportunity to simultaneously override numerous relevant “checkpoints” including sensitivity to regulatory T cells, suppression by TGF-β, and immune regulation not only by CTLA-4 (because of the CD28-independence of Cbl-b^−/−^ T cells) but also, as we now report, immune regulation by the PD-L1/PD-1 pathway.

## Author Contributions

MF designed the experiments, carried out the studies, analyzed the data, and wrote the manuscript. EA designed the experiments and analyzed the data. RC designed the experiments, analyzed the data, and wrote the manuscript.

## Conflict of Interest Statement

The authors declare that the research was conducted in the absence of any commercial or financial relationships that could be construed as a potential conflict of interest.

## Abbreviations

Cbl-b, casitas B-lineage lymphoma-b; PD-L1/PD-1, programmed death-ligand 1/programmed death-1; Tregs, regulatory T cells; MFI, mean fluorescence intensity; SHP-1/2, Src homology region 2 domain-containing phosphatase-1/2.
